# Evaluation of Warpage and Residual Stress of Precision Glass Micro-Optics Heated by Carbide-Bonded Graphene Coating in Hot Embossing Process

**DOI:** 10.3390/nano11020363

**Published:** 2021-02-01

**Authors:** Lihua Li, Jian Zhou

**Affiliations:** 1 Sino-German College of Intelligent Manufacturing, Shenzhen Technology University, Shenzhen 518118, Guangdong, China; lilihua@sztu.edu.cn; 2 School of Mechanical Engineering, Hefei University of Technology, Hefei 230009, Anhui, China

**Keywords:** carbide-bonded graphene, hot embossing, thermal effects

## Abstract

A newly developed hot embossing technique which uses the localized rapid heating of a thin carbide-bonded graphene (CBG) coating, greatly reduces the energy consumption and promotes the fabrication efficiency. However, because of the non-isothermal heat transfer process, significant geometric deviation and residual stress could be introduced. In this paper, we successfully facilitate the CBG-heating-based hot embossing into the fabrication of microlens array on inorganic glass N-BK7 substrate, where the forming temperature is as high as 800 °C. The embossed microlens array has high replication fidelity, but an obvious geometric warpage along the glass substrate also arises. Thermo-mechanical coupled finite element modelling of the embossing process is conducted and verified by the experimental results. Based on trial and error simulations, an appropriate compensation curvature is determined and adopted to modify the geometrical design of the silicon wafer mold. The warpage of the re-embossed microlens array is significantly decreased using the compensated mold, which demonstrates the feasibility of the simulation-oriented compensation scheme. Our work would contribute to improving the quality of optics embossed by this innovative CBG-heating-based hot embossing technique.

## 1. Introduction

Cost-effective mass production of precision micro-optics, such as microlens arrays (MLA), diffractive gratings and Fresnel lenses, has attracted attention in both academic and industrial fields. Amongst various fabrication techniques, hot embossing [1,2] and molding [3,4] are two types of net-shape replication methods to fabricate glass optics. Nevertheless, the applications of the conventional hot embossing and molding techniques are still limited due to low energy efficiency during production, since the bulk specimen, the whole mold assembly and the containing chamber are all heated to high temperature. Most of the heat energy is wasted in the heating-cooling cycles. Moreover, the lifespan of the precious molds could be severely reduced under cyclic thermal-mechanical loads.

A superior carbide-bonded graphene (CBG) network coating [5] first plays an important role as non-sticking protective coating in hot embossing and molding, because of its extraordinary mechanical properties and surface quality. The CBG coating’s feasibility of protecting the silicon mold from adhesion with softened glass, was confirmed by Peng’s work [6], where they successfully fabricated a Fresnel lens and microwells at molding temperature of 640 °C. In addition, the strong durability of the CBG coating at high temperature was experimentally validated. The protective role of CBG coating was also reported in other cases for molding of glass [7,8,9] and polymeric optics [10]. Nevertheless, in these studies, the CBG coating only served as protective coating, whereas the heat sources were still from traditional infrared radiation or induction.

Besides, the thin CBG coating can serve as the fast heating source because of its high electrical and thermal conductivity. Its fast heating capacity is strongly supported by that the CBG-coated zirconia substrate can achieve rapid thermal cycling in the range of 150–320 °C with a heating rate as high as about 50 °C/s, while a low power input of 48 W is required [11]. Therefore, a localized rapid surface heating approach, which is realized by taking advantage of the fast Joule heat generation performance of the CBG coating, has been developed to overcome the shortcomings of conventional hot forming techniques. Since only the specimen and the core molds are heated up, the required energy is drastically decreased. Hui et al. [12] successfully embossed high-quality microstructures on chalcogenide glass using a CBG-coated fused silica wafer, thus firmly demonstrated the feasibility of the localized rapid heating approach. Xie et al. [13] implemented replications of microchannel and microlens arrays onto poly(methyl methacrylate) (PMMA) substrates using a CBG-coated silicon mold. Furthermore, the application of the CBG-coated silicon mold realized rapid thermal cycling in injection molding, where the average heating rate was as high as 11.6 °C/s and polypropylene (PP) plate samples with uniform sizes in thickness were produced [14].

As these forming studies fabricated optics on chalcogenide glass and polymeric substrates, the highest forming temperatures were less than 300 °C. The hot forming of common inorganic glasses requires much higher temperature which is typically higher than 500 °C. In order to facilitate the CBG localized rapid heating into the hot embossing of glass optics, previously the authors first fully investigated the heating behaviors of the CBG-coated silicon wafer under different gaseous environments and voltage/current inputs [15]. We demonstrated that the surface temperature of the CBG-coated silicon wafer could reach as high as 1300 °C, which absolutely satisfies the glass forming requirement. Then the whole heating process was simulated using COMSOL and validated by experimental results [16]. Finally, successfully attempts were achieved in embossing microlens array on glass P-SK57 with great surface integrity and replication fidelity [17], where the CBG localized heating temperature was as high as 600 °C.

In sum, the above studies have greatly promoted the application of the CBG localized rapid heating into polymers and inorganic glasses hot forming techniques. Typically, in order to ensure the geometric deviation and residual stress of embossed products within acceptable quality demands, both uniform temperature distribution and slow cooling rate are required [18]. A warpage of only a few microns on the substrate could affect the accuracy of the precision optics [19]. However, the temperature distribution generated by CBG localized heating is non-isothermal, besides, the whole thermal history is very short compared to conventional bulk heating. Thus, a much higher temperature gradient would occur during both heating and cooling stages, thus generate more geometrical warpage and residual stress, resulting in a degraded embossing quality. For example, finite element analysis for simulating the heat transfer between CBG coating and PMMA, indicated that the temperature gradient in PMMA sample was drastic in localized rapid heating [20]. Significant residual stress phenomena in molded optics were observed when using CBG localized heating [17]. Moreover, when the CBG-based hot embossing is applied to inorganic glass, whose transition temperature is much higher than polymeric materials and chalcogenide glasses, the quality-degradation problem is expected to be more serious. Nevertheless, to the authors’ best knowledge, studies are still scarce addressing the geometric warpage and residual stress in CBG localized heating based hot forming, especially for cases of inorganic glasses.

Therefore, the geometrical warpage and residual stress issues in the CBG-based hot-embossing process urgently need to be addressed. In this paper, we perform replication of a microlens array onto inorganic glass using the CBG-based hot-embossing technique, where the forming temperature is as high as approximately 800 °C. Then the warpage and residual stress are carefully analyzed through experimental and simulation approaches, thus finally enables compensation of the mold design to minimize the warpage.

## 2. Materials and Methods

### 2.1. Carbide-Bonded Graphene Coating Based Hot Embossing Process

When an electrically conductive material is subjected to a voltage *V*, the resultant current *I* generates a heat flow $\dot{Q}$ mainly by the Joule effect dependent on the electrical resistance *R*, is given by $\dot{Q}=I^{2}R$.

**Figure 1:**
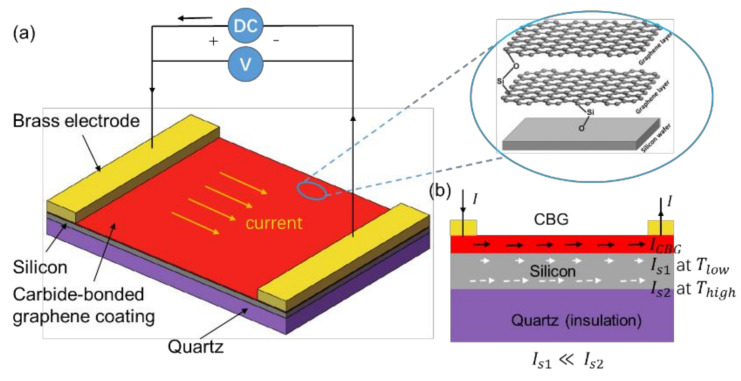
(**a**) Illustration of CBG Joule heating on silicon substrate, (**b**) current distribution between CBG coating and silicon substrate at low and high temperature.

Herein, a thin layer of CBG coating deposited on the intrinsic silicon wafer (Figure 1a), is designed to effectively generate surface Joule heat, by taking advantage of the CBG coating’s superior electrical conductivity (as high as $1.98\times 10^{4}\;S/m$) and thermal conductivity (1200 W/m·K on ceramic) [5]. An intrinsic silicon wafer is used as the substrate mainly for two purposes. Firstly, microstructures can be fabricated onto silicon via various methods, such as etching or ultra-precision machining. Secondly, when the silicon wafer is heated up to a certain temperature, its resistance drops drastically to a small value [21], even less than that of the CBG coating (Figure 1b), thus generating greater Joule heating power.

### 2.2. Deposition Method of Carbide-Bonding Graphene Coating

**Figure 2:**
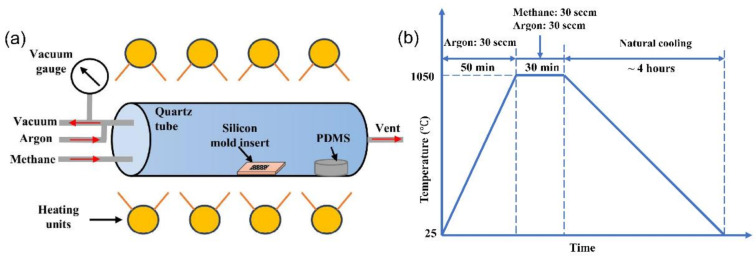
(**a**) CVD experimental setup for CBG deposition; (**b**) deposition process parameters as a function of coating time.

The deposition of the CBG coating on the silicon mold insert is realized by using atmosphere pressure chemical vapor deposition (APCVD), referring the reported procedure in ref. [5]. Concerning the complexity of using GPSO_3_H nanopapers, herein methane is selected as the main carbon source, and the silicon source is 99.8% pure solid polydimethylsiloxane (PDMS). Figure 2 presents the CVD deposition experimental setup and the deposition process parameters as a function of time. Prior to the coating process, the silicon mold and 2.0-g PDMS are placed in a quartz tube furnace with a diameter of 5.08 cm and a length of 0.6 m. The tube is first vacuumed to remove air and then purged with argon. After that, the temperature of quartz tube is heated up at a rate of 20 °C/min under argon flow at 30 sccm. Once the temperature reaches the suitable coating temperature of 1050 °C, methane is introduced at 30 sccm, together with the inert gas flow at 30 sccm. The tube temperature is held at 1050 °C for 30 min to allow coating growth. The silicon related radicals such as Si-O, O-Si-O, Si-C and C-Si-C, react with the carbon radicals, meanwhile the coating is gradually deposited on the silicon substrate layer by layer. The thickness of the deposited coating is mainly determined by the duration of this stage. After that, all gas flows are turned off, and then the tube is cooled down to the room temperature by natural cooling. Finally, the deposited silicon mold insert is taken out and washed with water and acetone for the removal of any ash on the surface.

### 2.3. Schematic of CBG-Based Hot Embossing Process

**Figure 3:**
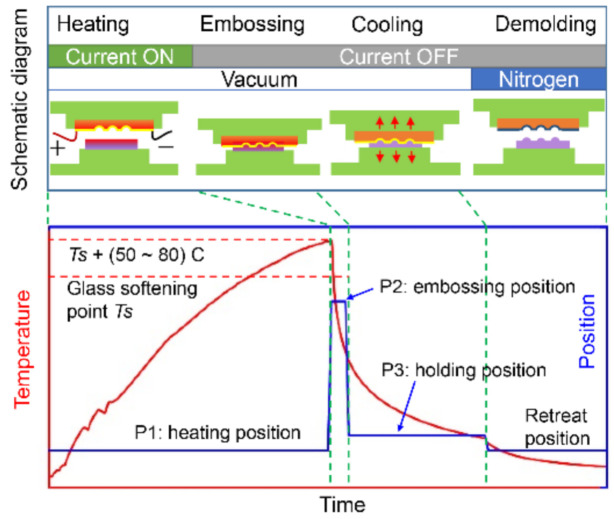
Schematic diagram of the CBG-based hot embossing process, and time history plots of temperature and position of the silicon mold.

The CBG-based hot embossing includes heating, embossing, cooling and demolding steps (Figure 3). The CBG-coated silicon mold is intended to be very close to the glass sample during the heating stage, so that the glass sample can be heated effectively mainly through gap conduction and radiation. The reason why the silicon mold does not contact the glass at evaluated temperature, is that when electrical current flows through a hot glass surface above the transition temperature [22], bubbles arise and accumulate within the glass, resulting in a non-acceptable defect. Since the glass’s surface temperature is hard to measure, the temperature of the silicon mold should be 50~80 °C higher than the softening point $T_{s}$ of the specific glass type before embossing. Here we use the relative temperature in Figure 3, because the value of $T_{s}$ is variable depending on the glass type. A thermocouple beneath the silicon mold is employed to monitor the temperature of the silicon mold. The heating process is performed under vacuum condition of 5 Pa. Once the desired temperature is reached, the current is turned off, and the silicon mold is moved downwards to quickly compress the softened top layer of glass within seconds. The embossing position is held for about 20 s to avoid immediate springback of the embossed structures. Once the current is off, the temperature of both the silicon mold and glass drop fast. When the temperature of the silicon mold drops below the glass transition temperature, the embossing force is released but a small force maintains the contact between glass and silicon mold so as to reduce the temperature gradient. When the monitored temperature is below 200 °C, nitrogen is purged to remove the vacuum condition, and the chamber is opened to demold the embossed sample.

### 2.4. Embossing Process and Experiment Setup

**Figure 4:**
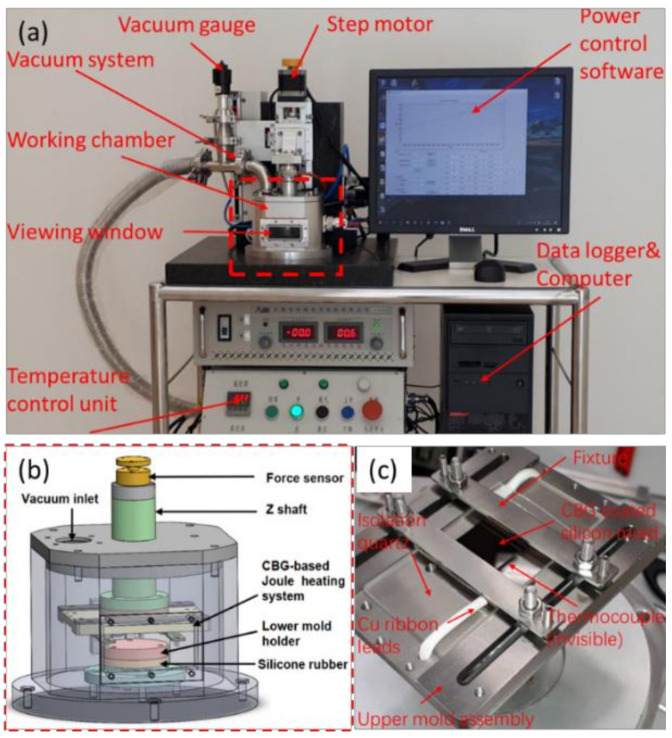
Hot embossing apparatus: (**a**) main components, (**b**) working chamber, (**c**) upper assembly with CBG-coated silicon mold.

**Figure 5:**
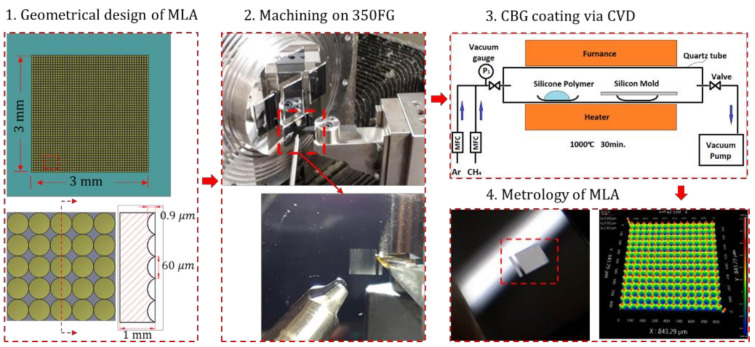
Fabrication process of CBG-coated silicon wafer mold with MLA.

A tailored hot embossing apparatus was constructed (Figure 4), mainly composed of the core CBG-coated silicon mold and other functional supporting components. Figure 4c shows the core CBG-coated mold assembly, where two Cu ribbon leads are used to apply the voltage input. A thick quartz plate, together with two small quartz bars, is used to electrically and thermally isolate the silicon mold from the surrounding bodies. Preparing the CBG-coated silicon mold mainly includes creating MLA on bare intrinsic silicon, and the deposition of the CBG coating (Figure 5). Single-point diamond turning (SPDT) performed on Moore Nanotech 350FG, is employed to fabricate a closely packed plano-concave MLA on a monocrystalline silicon wafer. The geometrical design of the MLA is presented in Figure 5. Herein one row of lenslets are machined by two separate steps using the broaching mode, so as to mitigate the excessive *Z*-axis acceleration at the sharp-edge boundaries. The fabricated microlens array has a form error as small as 30 nm in PV, and surface roughness of 2 nm in Ra. Then a thin layer of CBG coating with approximate thickness of 300 nm is deposited onto the fabricated mold by chemical vapor deposition (CVD). Finally, the CBG-coated silicon mold is mounted in the embossing device. The material of the glass wafer is N-BK7 from Schott Glass Technologies (Suzhou, China) Co., Ltd., whose glass transition temperature $T_{g}$ and softening point $T_{s}$ are 557 °C and 719 °C respectively. The glass dimensions are 12.7 mm in diameter and 5.0 mm in thickness.

### 2.5. Finite Element Simulation of Embossing Process

**Figure 6:**
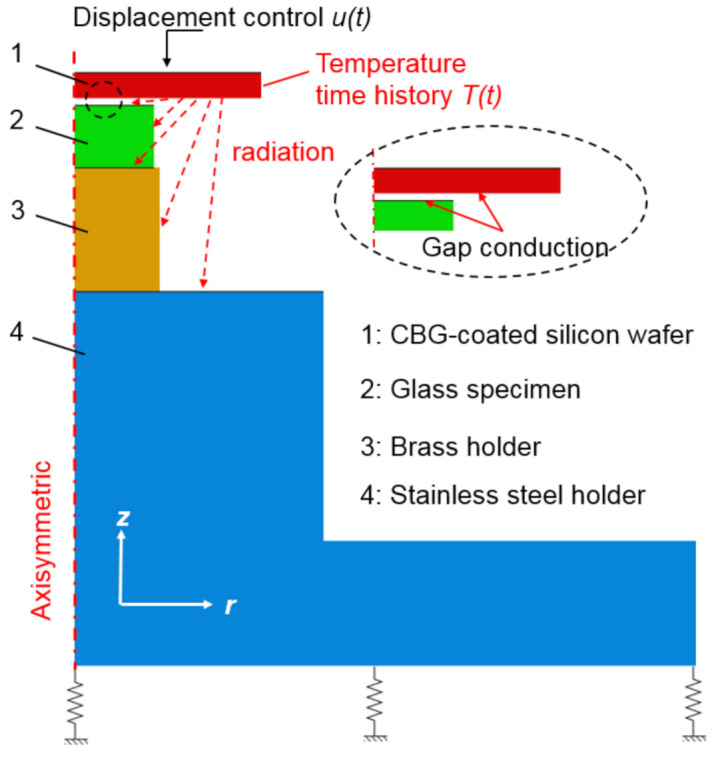
Axisymmetric finite element model for simulating the hot embossing process.

To predict the warpage and residual stress for the embossed product, a thermal-mechanical coupled finite element analysis is implemented in Abaqus. The influence of the MLA’s existence on the warpage and residual stress is negligible, so it is not included. To reduce the computational costs, the core parts are simplified as axisymmetric model (Figure 6). The bottom stainless-steel holder is linked to the ground with springs whose stiffness is determined as about 100 N/mm. The interfacial behavior between glass and silicon mold is modelled using the Coulomb friction model with a friction coefficient of 0.20. Time-dependent displacement and temperature history are applied to the silicon mold. Radiation from the silicon mold onto the glass sample, brass and stainless-steel holder are considered. Since most of the radiation is transmitted through N-BK7 glass, radiation can reach the top surface of brass holder. The gap conduction between glass and silicon mold is also included. Softened glass is modelled as a viscoelastic material by using the generalized Maxwell model in the Prony series [23]. The Williams-Landel-Ferry (WLF) equation is used to characterize the thermal-rheology behavior [24]. Additionally, structural relaxation using Tool–Narayanaswamy–Moynihan (TNM) model [25] is included to fully characterize the thermal expansion of the viscoelastic glass. These viscoelastic material parameters of N-BK7 glass can be obtained from previous studies [9,26]. The other parts are modelled as elastic deformable bodies.

## 3. Results

### 3.1. Characterization Results of the CBG Coating

**Figure 7:**
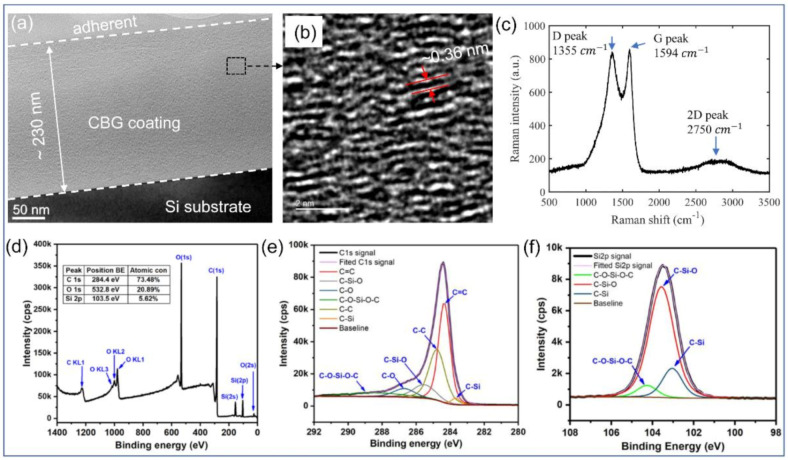
Characterizations of the CBG coating: (**a**) HRTEM image across the cross-section of the silicon mold insert, (**b**) higher magnification HRTEM image of a small region in (**a**), (**c**) Raman spectrum, (**d**) overall XPS spectrum analysis, (**e**) C_1s_ spectrum and (**f**) Si_2p_ spectrum.

Figure 7 presents some typical characterization results of the CBG coating, including high resolution TEM (HRTEM), Raman spectrum and X-ray photoelectron spectroscopy (XPS) analysis. First, high resolution TEM observation (JEM-2100F, Japan) along the cross-section of the coated silicon mold, is made to present the spatial structure of the deposited coating (Figure 7a). The thickness is determined as about 230 nm, with more than 600 layers of graphene in total. From a higher magnification image of a small area (Figure 7b), it is clearly observed that the graphene layers are uniformly aligned in-plane and well stacked in the depth direction though some disorders exist. 

Then Raman spectroscopy and XPS are combined to determine the bonding structure. The Raman spectrum of the CBG coating is obtained using 532 nm laser (LabRam HR Evolution, France). As shown in Figure 7c, three typical characteristic peaks of graphene are identified, which are D peak at 1355 cm^−1^, G peak of 1594 cm^−1^ and 2D peak 2750 cm^−1^. Further, the ratio of I_D_/I_G_ is determined as 0.98 and I_2D_/I_G_ is 0.22. From the overall XPS survey spectrum (Figure 7d), the CBG coating is composed of three main elements carbon, oxygen and silicon with atomic concentrations of 73.48%, 20.89% and 5.62% respectively. Figure 7e shows the C_1s_ spectrum, in which the C=C and C-C bonds are located at 284.4 eV and 284.8 eV respectively. In this case, the sp^2^/sp^3^ ratio is estimated to be 1.4, indicating that the CBG coating is mainly composed of sp^2^ carbon sites. Moreover, the C-Si bond appears at a binding energy of 283.7 eV, and the C-Si-O bond is at 285.5 eV. The weak peak near 286.7 eV is assigned to the C-O bond, which implies that the surface of the CBG coating is slightly oxidized due to exposure to the air. The left peak appearing at 288.3 eV is assigned to the C-O-Si-O-C bond. From the Si_2p_ spectrum, the binding energies of C-Si, C-Si-O and C-O-Si-O-C bonds are located at 103.0 eV, 103.6 eV and 104.3 eV respectively. A few previous studies [5,27,28] have confirmed that the carbon coatings dominated by sp^2^ sites exhibit low external stress, excellent mechanical and electrical performances. Besides, the existence of sp^3^ sites contributes to the high toughness of the CBG coating. Benefiting from these merits, the CBG coating serves an extraordinary protective coating for enhancing the embossing performance and elongating the lifespan of the silicon molds.

The resistance of the CBG-coated silicon mold *R*, is measured as 250 Ω by using four-point probe method. Since the intrinsic silicon substrate is nearly insulator (>10 KΩ) at room temperature, the electrical conductivity of CBG coating is calculated as σ=L/RA=L/RWh neglecting the effect of substrate, where *L* and *W* are the length and width of the mold respectively, *R* is the measured resistance and *A* is the cross-section area. Here *L* = *W* as the mold’s shape is square, and the thickness of the CBG coating *h* is about 230 nm from HRTEM measurement. Then we get $\sigma =1/Rh=1.74\times 10^{4}\;S/m$, which is very close to the reported value of $1.98\times 10^{4}\;S/m$ [5].

### 3.2. Embossed Microlens Array with Curvature

**Figure 8:**
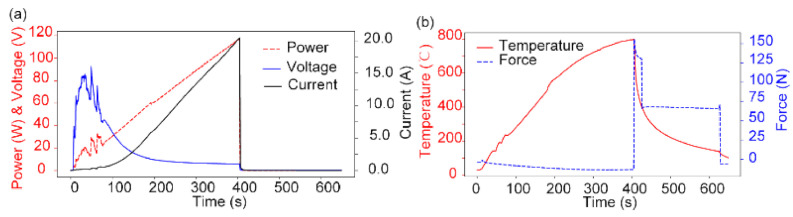
Time history plots of process parameters: (**a**) applied voltage, current and power, (**b**) temperature of silicon mold and applied force.

According to the embossing temperature requirement, the maximum temperature on silicon is expected as about 800 °C, which is realized by a ramp power control with a maximum power of 120 W within 400 s (Figure 8a). A small gap between glass and silicon is set as 0.1 mm before embossing. The embossing process is finished within 20 s with a maximum compression force of about 150 N (Figure 8b). Noticeably, the whole heating process only consumes approximately 24 KJ to achieve a very high temperature of 800 °C, which is almost less than 1% of the total energy required in the conventional embossing process [2], thus strongly proves the energy-saving advantage of the localized heating method. Moreover, the duration of the whole embossing process only takes about 600 s, also much more efficient than the conventional processes.

**Figure 9:**
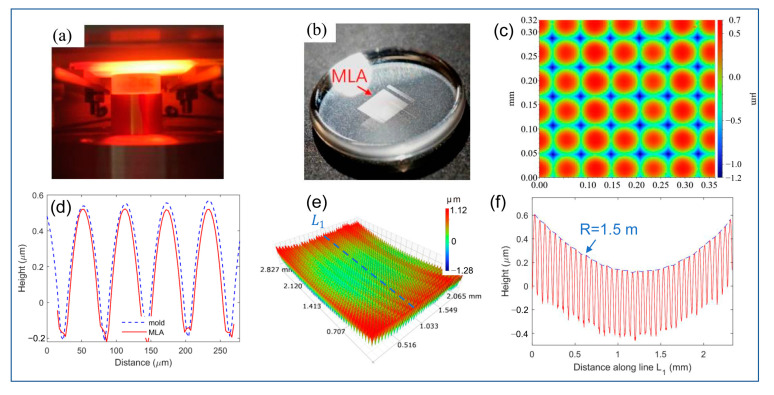
(**a**) Photo of the CBG-heating-based hot embossing in process, (**b**) the embossed MLA on N-BK7 glass wafer, (**c**) contour of a small region in the center of the embossed MLA, (**d**) profile comparisons of the selected MLA in (**c**) with designed mold profile, (**e**) contour of a large region on the MLA, (**f**) the profile scan of the contour in (**e**) along line L_1_.

By using the CBG-based hot embossing process (Figure 9a), the embossed MLA on the glass wafer is presented in Figure 9b. The feasibility of this hot embossing process is demonstrated for inorganic glass with a very high molding temperature. When we evaluate only a small region (0.35 mm × 0.32 mm) in the center of the MLA in Figure 9c, from the height contour captured by white-light interferometer Wyko NT8000, the selected MLA is uniformly distributed, and the heights of all peaks are almost the same. As shown in Figure 9d, the profile of the selected MLA well agrees with that of the mold, with an error less than 30 nm, which strongly demonstrates the microstructures filling feasibility. However, when a large area (2.82 mm × 2.06 mm) of the embossed MLA is scanned in Figure 9e, an obvious macroscopic geometric warpage is observed. Figure 9f more clearly presents the warped profile scan of the embossed microlens along the marked line L_1_ along the glass wafer. In detail, the curvature of the arc in Figure 9f is calculated using the equation $R=L^{2}/8\delta$, where *L* = 2.33 mm is the chord length of the arc, and δ=0.45 μm is the sagitta, and then *R* = 1.50 m is estimated. The upward bent profile directly indicates that significant warpage is introduced into the glass wafer due to the non-isothermal heat transfer process.

### 3.3. Evolution of Geometrical Warpage and Residual Stress

**Figure 10:**
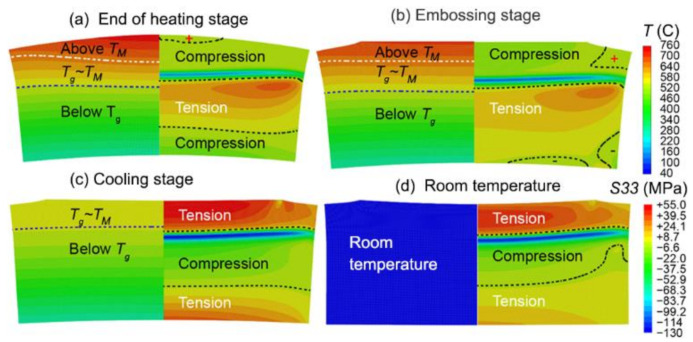
Illustration of temperature (left part of the contour) and hoop stress (right part of the contour) distribution contours at four different stages of embossing (glass viscosity = 10^8.0^ Pa. s at $T_{M}$); (**a**) End of heating stage; (**b**) Embossing stage; (**c**) Cooling stage; (**d**) Room temperature.

**Figure 11:**
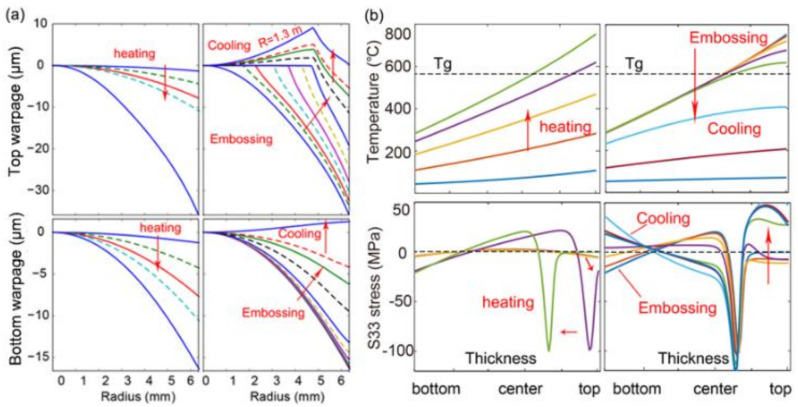
(**a**) Top and bottom warpage’s evolution, (**b**) temperature and hoop stress along the center line from bottom to top during the whole embossing process.

During the heating stage, as the temperature of the top surface is much higher than the bottom surface (Figure 10a), the glass sample expands more than that of the bottom surface along the radial direction. As a result, the glass is bent upwards, so both the top and bottom warpage of the glass wafer are defined as negative (Figure 10a). With the temperature gradient increasing during the heating stage, both bending curvatures keep increasing. During embossing, the contact area on the top surface of the glass is flattened and gradually increases while the glass flows outward in the tangential direction (Figure 10b). If only part of the top surface is embossed, a bump could exist finally. Since the bottom surface of glass is still in solid state and not easy to be deformed, the shape of the bottom surface does not obviously change. During the cooling stage, the top layer of glass is cooled faster than the surroundings, so the glass sample turns out to be bent downwards, resulting in that both the top and bottom warpages gradually change from negative to positive (Figure 10c,d). The final warpage curvature of the top surface of glass is determined as 1.3 m from the simulated result (Figure 11a), which is very close to the experiment value of 1.5 m. The bottom warpage is about 1.0 μm with curvature of 20.0 m, which is much less than that of the top surface.

The thermal stress is very low during the early heating stage since the whole glass is still in a solid state and expands uniformly. When the top layer of glass is first heated above the transition temperature $T_{g}$, the top layer of glass changes into a viscoelastic state and expands at the rate of $\alpha_{l}\;$($\alpha_{l}=54\times 10^{-6}/℃$, the coefficient of thermal expansion in liquid state) which is much greater than $\alpha_{g}$ ($\alpha_{g}=8.2\times 10^{-6}/℃$, the coefficient of thermal expansion in glassy state). The effective thermal strain increases intensely, thus drastically increases the compression stress on the top layer. With further increasing temperature, the compression stress gradually decreases to a smaller value because of viscoelastic stress relaxation. The significant compression stress transfers from the top layer to the central layer since the region above the transition temperature moves down. However, during the cooling stage, both the stress within both the top and bottom layers of the glass turn out to be tensile, because these layers are cooled much faster than the middle region. This phenomenon is particularly obvious for the top layer. In summary, significant compression stress is induced during the heating stage, meanwhile obvious tension stress is induced during the cooling stage (Figure 11b).

**Figure 12:**
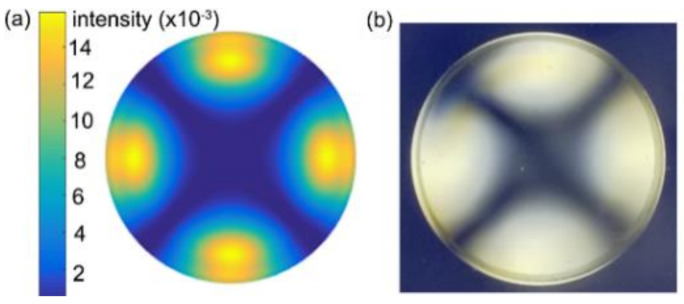
The birefringence optical retardation contours of the embossed glass from simulation (**a**) and experimental measurement (**b**).

The magnitude of the residual stress can be estimated by the birefringence retardation caused by the difference between the principal stresses [29]. Along the thickness of the glass sample, the birefringence retardation δ is given by $\delta =C\int_{0}^{h}\left(\sigma_{11}-\sigma_{33}\right)dz$ [30], where *h* is the thickness of the glass sample which is 5 mm, and *C* is the photoelastic constant estimated as 2.17 /TPa. The simulated and experimental light intensity show good agreement in patterns as shown in Figure 12.

### 3.4. Decreasing Top Warpage through Curvature Compensation

It has been well accepted that compensating the mold design before forming experiments is a time and economic saving approach to improve the geometric profile accuracy of the embossed/molded optics [31]. In order to decrease the top warpage, direct compensation on the replication mold’s geometrical profile is implemented lastly. As discussed above, the final glass sample is bent downwards, so a concave profile should be adopted for the top silicon mold. The compensation scheme is not applied to the bottom surface since its warpage is relatively small. The curvature of the concave profile $R_{c}$ is given by $R_{c}=0.25D^{2}/2h$, where *D* is the aperture and *h* is the sag.

**Figure 13:**
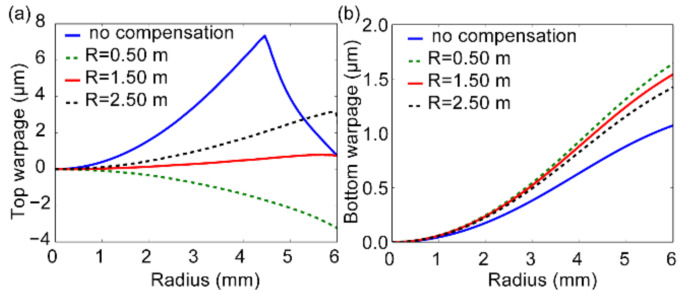
The newly obtained top (**a**) and bottom (**b**) warpage plots under different compensation curvature values.

**Figure 14:**
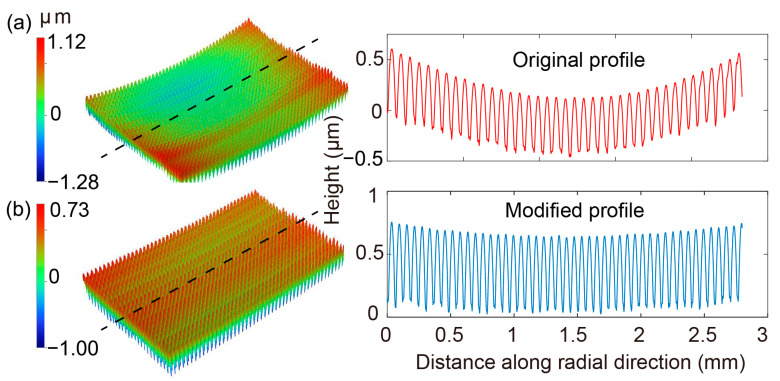
The warpage of the newly embossed MLA (**b**) using the modified silicon mold, with comparison to the original warpage profile (**a**).

Three curvature trials of 0.50 m, 1.50 m, 2.50 m are tested here. As indicated from the newly obtained warpage curves (Figure 13a), the choice of the compensation curvature would significantly change the final pattern of the top warpage. Meanwhile, the compensation applied on the top mold, does not much affect the bottom warpage (Figure 13b), which is beneficial in this case. When a flat compensation curvature of 2.50 m is adopted, the warpage is obviously decreased but still doesn’t meet the expected value. However, if the curvature is too small to be appropriate ($R_{c}\;$= 0.50 m), negative warpage would be generated. Thus, a proper compensation curvature should be determined by using trial and error method. Herein, from the trial simulation results, when a compensation curvature of 1.50 m is adopted, the resultant warpage is very small, with a curvature of 16.3 m, which can be regarded as a good choice. Eventually, when MLA is replicated by using the modified silicon mold with a curvature of 1.50 m, the warpage is evidently reduced in comparison to the original scan profile (Figure 14).

## 4. Conclusions

We successfully employ the localized rapid heating of a thin carbide-bonded graphene coating into the hot embossing of microlens arrays on inorganic glass N-BK7 substrate, where the required forming temperature is as high as 800 °C. The application span of this newly developed hot embossing technique is thus widely extended. The embossed MLA possesses high replication fidelity, however, a significant geometric warpage on the glass wafer substrate is introduced because of the non-isothermal heat transfer. Thermo-mechanical coupled finite element simulation on the embossing process not only reveals how the geometrical warpage and residual stress evolve, but also well predicts the curvature of the warpage. Based on trial and error simulations, an appropriate compensation curvature is determined and adopted to modify the geometrical design of the silicon wafer mold. The warpage of the re-embossed microlens array using the compensated mold is significantly decreased, which demonstrates the feasibility of the simulation-oriented compensation scheme. Compared to the conventional hot forming processes using bulk heating, the CBG-heating-based hot embossing shows prevailing advantages in saving energy and enhancing efficiency. Our work would help promote the quality of the embossed optics using this newly developed technique. Further investigation on the coating’s lifespan and degradation mechanism under the harsh thermal history would be included in the future work.

## Data Availability

The data presented in this study are available on request from the corresponding author.
